# A new bacterial consortia for management of Fusarium head blight in wheat

**DOI:** 10.1038/s41598-024-60356-4

**Published:** 2024-05-02

**Authors:** Vishnukiran Thuraga, Farideh Ghadamgahi, Fantaye Ayele Dadi, Ramesh Raju Vetukuri, Aakash Chawade

**Affiliations:** Department of Plant Breeding, Swedish University of Agricultural Sciences, Växtförädling, Box 190, 234 22 Lomma, Sweden

**Keywords:** Wheat, Chlorophyll fluorescence, Bacterial consortium, *Fusarium*, DON, Plant sciences, Plant breeding, Plant stress responses

## Abstract

Fusarium head blight (FHB) is a significantly important disease in cereals primarily caused by *Fusarium* species. FHB control is largely executed through chemical strategies, which are costlier to sustainable wheat production, resulting in leaning towards sustainable sources such as resistance breeding and biological control methods for FHB. The present investigation was aimed at evaluating newly identified bacterial consortium (BCM) as biocontrol agents for FHB and understanding the morpho-physiological traits associated with the disease resistance of spring wheat. Preliminary evaluation through antagonistic plate assay and in vivo assessment indicated that BCM effectively inhibited Fusarium growth in spring wheat, reducing area under disease progress curve (AUDPC) and deoxynivalenol (DON), potentially causing type II and V resistance, and improving single spike yield (SSPY). Endurance to FHB infection with the application of BCM is associated with better sustenance of spike photosynthetic performance by improving the light energy harvesting and its utilization. Correlation and path-coefficient analysis indicated that maximum quantum yield (QY_max) is directly influencing the improvement of SSPY and reduction of grain DON accumulation, which is corroborated by principal component analysis. The chlorophyll fluorescence traits identified in the present investigation might be applied as a phenotyping tool for the large-scale identification of wheat sensitivity to FHB.

## Introduction

Wheat is a major food crop with an annual production of 760 megatons and nourishment source for ~ 40% of the world population, accounting for 20% of daily protein intake. World wheat production was reaching 3.5 × 10^4^ hg/ha, harvested from 2.2 × 10^8^ hectares^1^. Wheat production is threatened by crop diseases such as pathogenic fungal infections, which, under favorable conditions, can lead to yield losses of up to 60%. FHB also called scab, head scab or ear blight, is one of the most devastating diseases of wheat, barley and maize, with serious impacts on the yield, besides the deteriorating effects on human and animal health. FHB is caused by diverse *Fusarium* species, viz*., F. graminearum, F. avenaceum, F. culmorum, F. poae, and F. sporotrichioides* and among all the species, *F. graminearum* Schwabe (teleomorph = *Gibberella zeae* (Schw.)) Petch is the most widespread pathogen across the world^2–5^. The FHB outbreaks during the late nineteenth century in the USA led to whooping losses of $4.8 billion in wheat production, in addition to overall direct losses of ~ $1.3 billion^5,6^. The International Maize and Wheat Improvement Center (CIMMYT) has listed FHB as one of the most destructive to global wheat production^7–9^. Inhabiting of healthy spikes by Fusarium pathogen results in the production of mycotoxins in the grains, ingestion of which leads to myco-toxicosis, and other adverse mutagenic, teratogenic, estrogenic effects in human and animal bodies^7,10–13^. Among all the mycotoxins, DON is an potential protein synthesis inhibitor with resistance to heat, atmospheric pressure, weak acid, long term storage^14,15^. Besides different strategies (crop rotation, tillage practices, using resistance cultivars) used to control FHB incidence, fungicides continue to play an indispensable role in controlling Fusarium head blight under field conditions; however, increasing the dependency on fungicides is discouraged in terms of high production cost, potential environmental, public health risks^16^. Targeting the FHB with its natural enemies offers an additional strategy of biocontrol towards the integrated management of FHB, with a lesser risk of pest resistance development and environmental hazards^17,18^. Fungi, viruses, and plant growth-promoting rhizobacteria (PGPR) are among the potentially beneficial microorganisms being used as biocontrol agents (BCAs) with more opportunities for enrichment and new resource identification^19,20^. Members of the *Bacillus* genus have been reported to be one of the important potent PGPRs in a variety of plants, and this genus is also known for their use as BCAs against several pathogenic fungi^21–23^. One of the strains of *Bacillus subtilis*, *Pseudomonas* sp., *Cryptococcus* sp. and *C. nodaensis* exhibited a reduction in Fusarium head blight infection by 25% in wheat, but the grain DON content was not varied significantly^24–27^.

Photosynthetic carbon assimilation is the yield-determining factor contributed by foliar and green non-foliar organs such as stems and spike/panicles. Previous studies revealed that wheat ear is one of the important contributors to the total carbon assimilation by accounting for 10–59% of the total grain weight depending on the genomic constitution and environmental conditions^28,29^. There is increasing recognition that microbial growth within the plants can influence the light-dependent photosynthetic reactions, viz., *Pseudomonas* sps. had partially rescued the PSII efficiency under heavy metal stress and downregulated the electron transport under salt stress with protective stomatal closure in tomato plants^30,31^. Moreover, few studies have emphasized the idea that there is phenotypic evidence that photosynthetic parameters are altered to combat different fungal pathogens in plants^32^.

Nevertheless, previous research on BCAs unraveled potential endophytes for controlling FHB infection^33–35^. In the present study, we isolated six new bacterial endophytes from wild finger millet and evaluated their influence on FHB infection DON accumulation as a consortium in relation to SSPY. We also investigated the spike fluorescence and grain morphology to obtain a comprehensive understanding of how plant-BCA interactions are influencing the FHB disease and yield. Hence, the present investigation was aimed at (i) Evaluating the newly identified bacterial consortium in FHB control in spring wheat. (ii) To delineate the grain morphological and spike photosynthetic characters on SSPY and FHB-associated DON accumulation.

## Results

### Fusarium compatibility and BCM antagonistic assay

**Figure 1:**
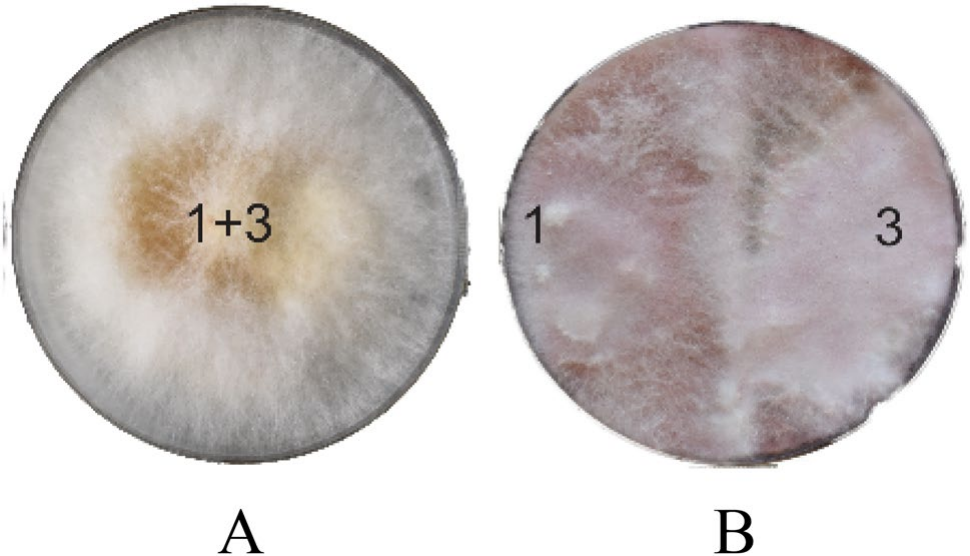
Compatibility test for two isolates of *Fusarium graminearum* (numbers 1 and 3 represent *Fusarium graminearum* isolates 1 and 3).

**Figure 2:**
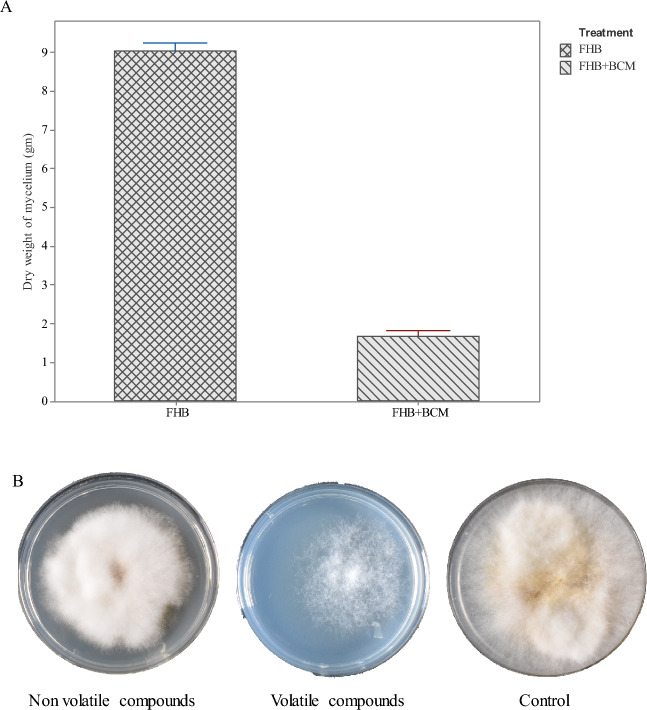
Antagonistic assay of BCM induced reduction in mycelial growth (The error bar represents standard error mean).

Mycelial growth of two Fusarium isolates on potato dextrose agar plates showed no zone of separation when plated in the center (Fig. 1A) or at one cm distance from the edge of the potato dextrose agar plate (PDA) (Fig. 1B), indicating a good compatibility between the two Fusarium isolates used in the study. The antagonistic activity of BCM against Fusarium is evident from the plate assay of volatile compounds (VOCs), non-volatile compounds (NVOCs) and mycelial dry weight evaluations. Mycelial growth was reduced by 81.6% in PDA liquid media samples (Fig. 2A), followed by VOCs (35% reduction) and NVOCs (24% reduction) (Fig. 2B).

### Greenhouse evaluation

#### Disease evaluation and single spike yield

**Figure 3:**
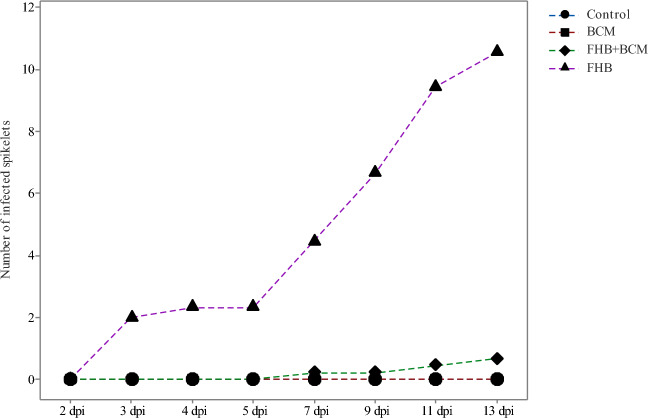
Progression of FHB infection across the four treatments from 2 to 13 dpi.

**Figure 4:**
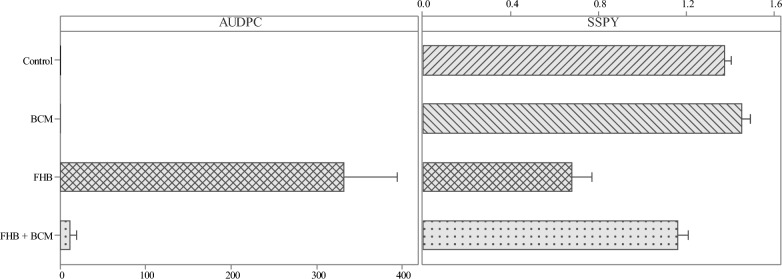
Interval plot of AUDPC and SSPY (g/spike) across the evaluated treatments. The bar represents standard error of the mean.

Application of BCM under controlled growth conditions resulted in a significant reduction in FHB infection and 47% reduction in grain DON accumulation (*P* < *0.001*). Symptoms of FHB infection were visible on two days post-inoculation (dpi) in untreated condition (FHB treatment), whereas BCM treatment (FHB + BCM) resulted in delayed onset of symptoms at seven dpi (Fig. 3). AUDPC and SSPY indicated significant differences between treatments (*P* < *0.001*) and application of BCM resulted in significant improvement in SSPY (71%) and reduction of AUDPC (96%) in FHB + BCM treatment (Fig. 4). Application of BCM resulted in a marginal improvement in grain yield of BCM (6%) in comparison with the control.

#### Spike fluorescence characters

Spike fluorescence characters varied significantly between the treatments, time points and significant interaction was noted for treatments × time points (Table 1). Spike fluorescence deteriorated with the progression of FHB infection and application of BCM resulted in the retention of spike fluorescence. Maximum fluorescence (F_m_) was varied from 211 (FHB) to 262 (FHB + BCM), while variable fluorescence (F_v_) ranged from 156 (FHB) to 206 (FHB + BCM). Quantum yield of PSII (F_v_/F_m_) and maximum PSII quantum yield (QY_max) were significantly increased under FHB + BCM in comparison to FHB. However, there are no significant differences among the control and BCM, FHB + BCM treatments. PSII operating efficiency (PSIIOPE) was found to be higher in control (0.680) followed by BCM (0.675), FHB + BCM (0.673) and least in FHB (0.652). The amount of open reaction centers (qL) varied significantly between treatments from 1.09 (FHB) to 1.31 (BCM), with a 4% increase by application of BCM. Quenching parameters viz*.,* non-photochemical (q_N_) and photochemical quenching(q_P_) did not vary significantly between the treatments; however the amount of q_N_ and q_P_ were marginally lower in BCM and FHB + BCM, compared to control and FHB. The non-photochemical quenching ranged from 0.188 (BCM, FHB + BCM) to 0.198 (control), where as photchemical quenching was varied from 0.829 (FHB) to 0.876 (control). The rate of steady state fluorescence decay (Rfd_Lss) was varied from 0.435 (FHB + BCM) to 0.527 (FHB) and 9% reduction noticed with application of BCM. The spike fluorescence was reduced from 2 to 13 dpi with progression of FHB disease, while the FHB + BCM maintained the effective spike fluorescence.

**Table 1:** Analysis of variance for spike fluorescence characters evaluated across treatments from 2 to 13 dpi.

	Mean Sum of Squares					Pr (> F)			
	Block	Treatment	Time point	Treatment × Time point	Error	Block	Treatment	Time point	Treatment × Time point
F_m_	263.991	44,738.701	7129.836	3808.250	1337.004	0.821^NS^	0.000***	0.000***	0.000***
F_o_	90.519	155.294	22.590	106.406	73.132	0.292^NS^	0.098^NS^	0.950^NS^	0.094^NS^
F_v_	174.220	41,604.487	6682.471	2804.609	931.057	0.830^NS^	0.000***	0.000***	0.000***
QY_max	0.000	0.224	0.050	0.035	0.008	0.988^NS^	0.000***	0.000***	0.000***
F_v_/F_m_	0.000	0.233	0.052	0.033	0.008	0.985^NS^	0.000***	0.000***	0.000***
q_N_	0.000	0.002	0.003	0.005	0.003	0.903^NS^	0.427^NS^	0.414^NS^	0.017*
q_P_	0.006	0.027	0.015	0.032	0.013	0.615^NS^	0.100^NS^	0.297^NS^	0.000***
qL	0.002	0.741	0.176	0.069	0.020	0.890^NS^	0.000***	0.000***	0.000***
Rfd	0.068	0.126	0.011	0.017	0.015	0.012^NS^	0.000***	0.645^NS^	0.328^NS^
PSIIOPE	0.0001	0.1055	0.0281	0.0266	0.0063	0.978^NS^	0.000***	0.0001***	0.000***

The area under curve (AUC) values for F_m_, F_v_, QY_max were significantly varied between treatments (Table 2). Significant imporovement in AUC of fluorescence parameters viz*.,* F_m_, F_v_, F_v_/F_m_, PSIIOPE, QY_max and qL and PSIIOPE were detected under FHB + BCM in comparision to FHB treatment (Table 2).

**Table 2:** Analysis of variance and multiple comparisions for area under curve values of spike fluorescence characters and morphology traits evaluated across treatments (Means followed by *different letters* among treatments represent significant difference at *P* < *0.05*).

	Trait	Mean				Mean sum of squares		Pr(> F)	
		Control	BCM	FHB	FHB + BCM	block	Treatment	block	Treatment
Spike fluorescence traits	F_m_	3400.67^a^	3375.67^a^	2722.67^b^	3423.44^a^	1993.53	1,035,456.78	0.983^NS^	0***
	F_o_	754	712.67	713.67	737.22	2243.11	3581.67	0.526^NS^	0.386^NS^
	F_v_	2646.78^a^	2663^a^	2008.89^b^	2685.89^a^	1769.19	971,556.99	0.979^NS^	0***
	F_v_/F_m_	10.09^a^	10.23^a^	8.66^b^	10.19^a^	0.00	5.15	0.998^NS^	0.001**
	QY_max	10.48^a^	10.58^a^	9.05^b^	10.54^a^	0.00	4.93	0.997^NS^	0.001**
	qL	16.4^a^	17.08^a^	14.1^b^	16.91^a^	0.05	17.13	0.967^NS^	0***
	q_N_	2.57	2.45	2.59	2.39	0.01	0.09	0.78^NS^	0.123^NS^
	q_P_	11.37	11.12	10.8	11.16	0.30	0.50	0.515^NS^	0.352^NS^
	PSIIOPE	0.6797^a^	0.674^a^	0.6501^b^	0.6724^a^	0.00	0.00	0.994^NS^	0.001**
	Rfd	6.48^a^	5.87^b^	6.84^a^	5.63^b^	2.54	2.78	0.003**	0.001**
Grain morphology traits	Area	11.36	12.04	10.99	12.75	1.044	5.443	0.748^NS^	0.228^NS^
	Length	5.55	5.92	5.65	5.81	0.038	0.247	0.747^NS^	0.147^NS^
	Width	3.09^a^	3.07^ab^	2.9^b^	3.21^a^	0.029	0.152	0.484^NS^	0.018*
	Thickness	2.58	2.64	2.49	2.81	0.012	0.168	0.826^NS^	0.061^NS^
	Mean width	2.88^ab^	2.9^ab^	2.75^b^	3.06^a^	0.019	0.148	0.686^NS^	0.048*
	Volume	22.98^ab^	24.5^ab^	20.35^b^	26.63^a^	12.742	62.746	0.552^NS^	0.047*
	Weight	0.2207	0.0348	0.027	0.0408	0.078	0.079	0.368^NS^	0.39^NS^
	Light	0.6142 ^a^	0.5672^b^	0.5993^a^	0.5872^ab^	0.000	0.004	0.689^NS^	0.037*
	Hue	26.04	26.06	26.25	25.61	1.087	0.649	0.638^NS^	0.845^NS^
	Saturation	0.4621	0.4696	0.4477	0.4732	0.002	0.001	0.179^NS^	0.461^NS^
	Roundness	0.6347	0.6233	0.6253	0.6541	0.000	0.002	0.619^NS^	0.034*
Spike and leaf morphology traits	FLT (mm)	0.1633^a^	0.14^b^	0.1588^a^	0.1511^ab^	0.0048	0.0009	0.00***	0.0254*
	SLA (cm^-2^ g^-1^)	242.33^c^	274^b^	243^c^	293^a^	346.75	5544.25	0.4075^NS^	0.00***
	SLW(g^-1^ cm^-2^)	0.0042^a^	0.0037^b^	0.0041^a^	0.0034^b^	0.000	0.00	0.3337^NS^	0.00***
	SPT (mm)	11.27^a^	10.9^a^	8.78^b^	10.59^a^	0.5016	10.99	0.5606^NS^	0.00***
	SPL (cm)	11.11	11.17	10.67	10.56	0.1438	0.8482	0.6471^NS^	0.0701^NS^

#### Grain morphology

Analysis of variance indicated significant variation between treatments for width, mean width, volume, light, and roundness (Table 2). Grain length, width, mean width, volume, and area were reduced under FHB treatment and application of BCM resulted in sustenance of grain length, width, mean width, volume and area of the grain in comparison with control and BCM treatments. Grain roundness was significantly reduced under FHB treatment and the application of BCM increased the grain saturation and roundness by 5–6%.

#### Flag leaf, spike morphology

Application of BCM resulted in significant variation in flag leaf thickness (FLT), specific leaf area (SLA), specific leaf weight (SLW), spike thickness (SPT) and significant interaction was detected between the treatments (Table 2). The FLT and SLW were reduced with the application of BCM by 14% and 12%, which is accompanied by significant increase in SLA by 13%. The SPT was marginally reduced under BCM treatment, while a significant reduction is noticed under FHB treatment and no significant variation was observed in spike length (SPL).

#### Correlation and path coefficient analysis

**Figure 5:**
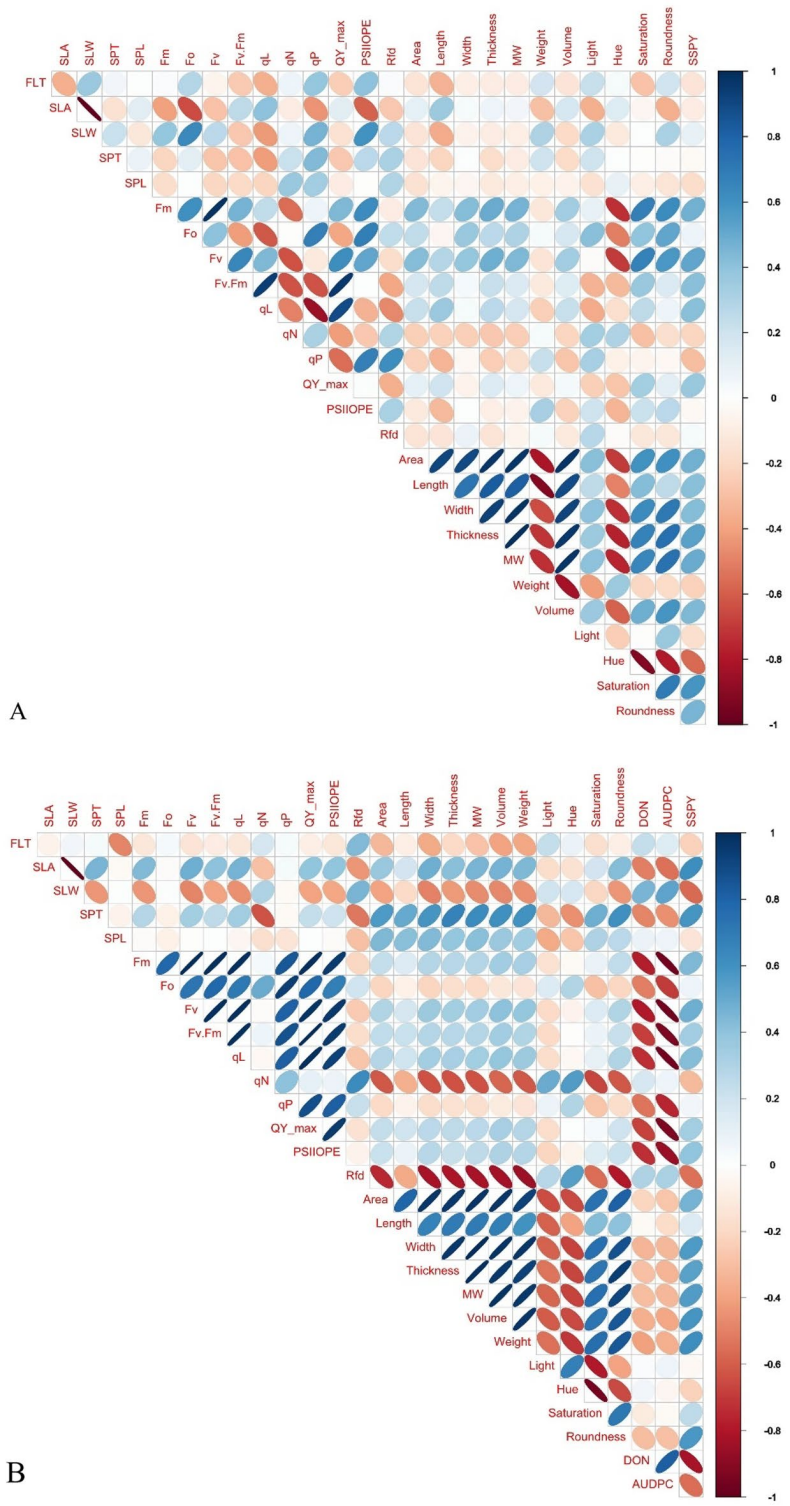
Correlation matrix of evaluated tratis with SSPY (**A**) and FHB infection traits (**B**) (FLT: Flag leaf thickness, SLA: Specific leaf area, SLW: Specific leaf weight, SPT: Spike thickness, SPL: Spike length, F_m_: Maximum fluorescence, F_o_: Minimum fluorescence, F_v_: Variable fluorescence, F_v_.F_m_: Quantum yield of PSII, qL: Fraction of open reaction centers, q_N_: Non-phochemcial quenching, q_P_: Photochemical quenching, QY_max: Maximum quantum yield of PSII, PSIIOPE: Operating efficiency of PSII, Rfd: Fluorescence decline ratio, Area: Grain area, Length: Grain length, Width: Grain width, Thickness: Grain thickness, MW: Grain mean width, Weight: Grain weight, Volume: Grain volume, Light: Grain light, Hue: Grain Hue, Saturation: Grain saturation, Roundness: Grain roundness, SSPY: Single spike yield, DON: Deoxynivalenol, AUDPC: Area under disease progress curve).

Pearson correlation analysis indicated SSPY has a significant positive correlation with fluorescence traits (F_m_, F_v_, QY_max, F_v_/F_m_, qL), grain morphological traits (width, volume, saturation) (Fig. 5A). AUDPC is negatively correlated with spike fluorescence characters (F_m_, F_o_, F_v_/F_m_, qL, q_P_, QY_max and PSIIOPE) and SSPY (Fig. 5B). DON accumulation in grain is negatively correlated with spike fluorescence characters, SSPY and SPT (Fig. 5B). Grain length, width, and thickness are negatively related to the AUDPC and DON accumulation in grain. A positive correlation was detected between grain roundness with length, width and area of the grain under uninfected and infected conditions. Spike fluorescence traits such as F_o_, F_m_ and F_v_ are positively related with grain roundness under uninfected conditions. SPT is positively correlated with grain morphology characters under FHB infected conditions (Fig. 5B). SPT, QY_max and roundness had direct positive effects on the SSPY (Table 3A). SPT, QY_max had direct negative effects on DON (Table 3B).

**Table 3:** Path coefficient analysis of selected traits influencing SSPY (3A) and grain DON accumulation (3B).

3A	FLT	SLW	SPT	SPL	qN	QY_max	Roundness
FLT	** − 0.1640**	** − **0.0597	** − **0.0087	** − **0.0007	** − **0.0114	0.0408	** − **0.0321
SLW	0.0156	**0.0429**	0.0092	** − **0.0052	0.0030	** − **0.0067	0.0135
SPT	0.0038	0.0153	**0.0713**	0.0062	0.0155	** − **0.0191	** − **0.0012
SPL	** − **0.0003	0.0100	** − **0.0071	** − 0.0825**	** − **0.0311	0.0080	0.0120
q_N_	0.0007	0.0007	0.0021	0.0036	**0.0097**	** − **0.0040	** − **0.0016
QY_max	** − **0.0759	** − **0.0474	** − **0.0817	** − **0.0297	** − **0.1254	**0.3054**	0.0364
Roundness	0.0833	0.1336	** − **0.0071	** − **0.0620	** − **0.0710	0.0508	**0.4258**
Indirect effect on SSPY	0.0272	0.0525	** − **0.0932	** − **0.0878	** − **0.2204	0.0699	0.0270
Total effect on SSPY	** − **0.1369	0.0955	** − **0.0219	** − **0.1703	** − **0.2108	0.3753	0.4528

3B	FLT	SLW	SPT	SPL	qN	QY_max	Roundness
FLT	**0.305**	0.016	0.012	** − **0.149	0.054	** − **0.026	** − **0.027
SLW	0.005	**0.095**	** − **0.041	0.001	0.029	** − **0.038	** − **0.041
SPT	** − **0.014	0.156	** − 0.358**	0.025	0.223	** − **0.083	** − **0.217
SPL	** − **0.098	0.002	** − **0.014	**0.201**	** − **0.034	0.000	0.052
q_N_	** − **0.010	** − **0.017	0.036	0.010	** − 0.057**	** − **0.006	0.035
QY_max	0.045	0.208	** − **0.122	** − **0.001	** − **0.053	** − 0.523**	** − **0.108
Roundness	0.000	0.000	0.000	0.000	0.000	0.000	**0.001**
Indirect effect on DON	** − **0.072	0.364	** − **0.129	** − **0.115	0.219	** − **0.152	** − **0.305
Total effect on DON	0.233	0.459	** − **0.486	0.086	0.162	** − **0.676	** − **0.305

FLT: Flag leaf thickness, SLW: Specific leaf weight, SPT: Spike thickness, SPL: Spike length, q_N_: Non-phochemcial quenching, QY_max: Maximum quantum yield of PSII, Roundness: Grain roundness, SSPY: Single spike yield, DON: Deoxynivalenol.

Direct effect values are in [bold].

#### Principal component analysis

**Figure 6:**
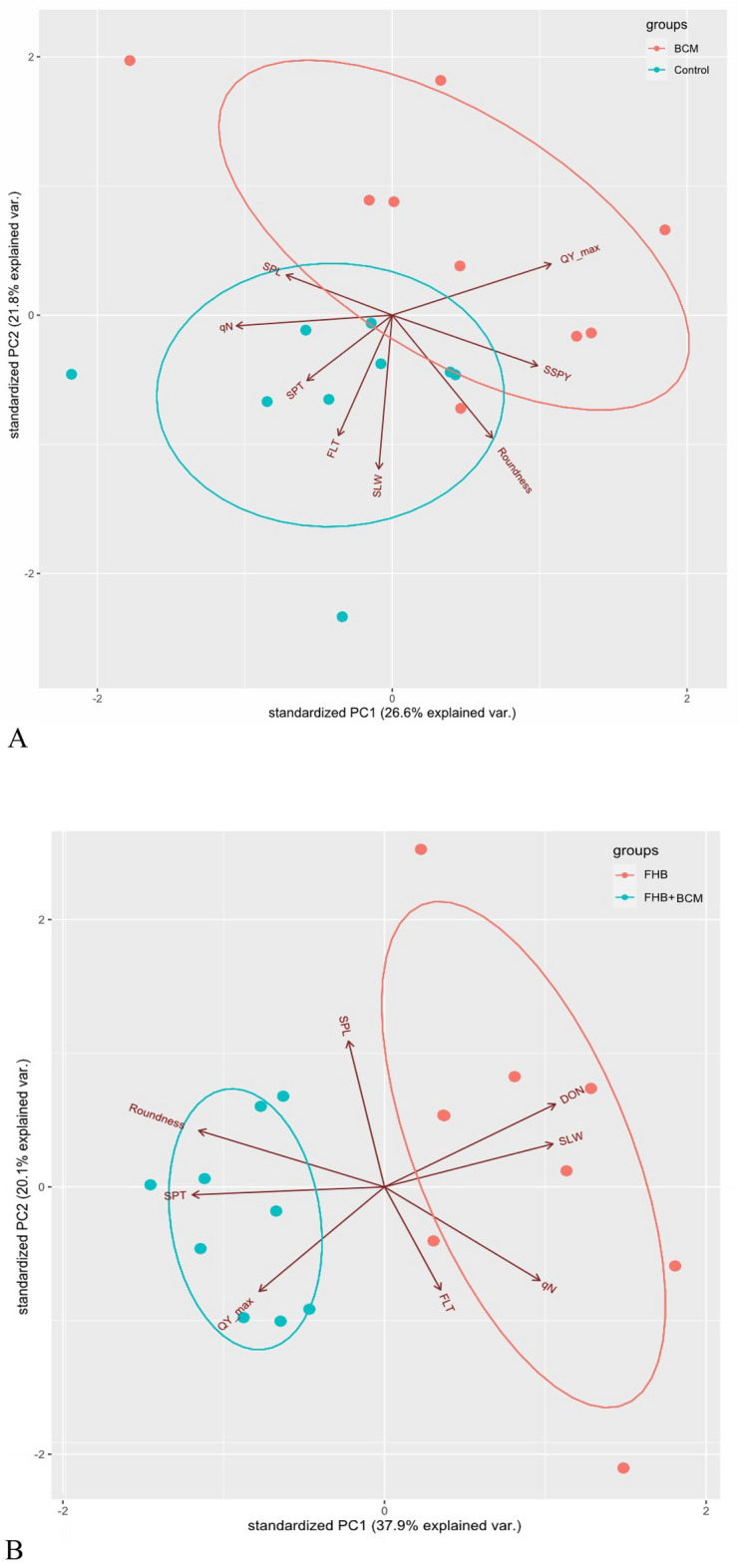
Loading plot of principal components evaluated under control and BCM (6A), FHB and FHB + BCM (6B).

Principal component analysis was executed to understand the relationship among the evaluated traits with SSPY and grain DON accumulation. The first three PCs exhibited a sum of 62% variation with the largest share from PC1 (26.6) and PC2 (21.8) (Table 4). QY_max, roundness and SSPY had the largest positive loadings on PC1, whereas roundness, SPL and QY_max had the largest positive loadings on PC2 (Fig. 6A). PC3 had large positive loadings from FLT. Under FHB infection, 75.6% of the variation is explained by the first three PCs, with a share of 37.9% from PC1, exhibiting the largest negative loadings from roundness, SPT, QY_max and positive loadings from DON, SLW (Table 4). PC2 explains 20.1% variation with high negative loadings from QY_max, qN and positive loadings from SPL, DON. PC3 had positive loadings from FLT, SPT, DON and negative loadings from SPL and q_N_ (Fig. 6B).

**Table 4:** Vector loadings and percentage variance explained by first three principal components.

	Trait	PC1	PC2	PC3
Control and BCM	FLT	** − **0.168	** − **0.475	0.391
	SLW	** − **0.042	** − **0.606	0.038
	SPT	** − **0.267	** − **0.258	** − **0.393
	SPL	** − **0.331	0.158	** − **0.489
	q_N_	** − **0.487	** − **0.042	** − **0.362
	QY_max	0.497	0.201	** − **0.166
	Roundness	0.313	** − **0.484	** − **0.216
	SSPY	0.455	** − **0.199	** − **0.497
	Eigenvalue	2.12676	1.7464	1.08405
	Cumulative variance%	26.6	48.4	62.0
FHB and FHB + BCM	FLT	0.135	** − **0.409	0.553
	SLW	0.404	0.171	0.015
	SPT	** − **0.462	** − **0.031	0.323
	SPL	** − **0.086	0.579	** − **0.382
	q_N_	0.373	** − **0.371	** − **0.357
	QY_max	** − **0.301	** − **0.415	** − **0.435
	Roundness	** − **0.446	0.224	0.191
	DON	0.411	0.329	0.301
	Eigenvalue	3.03159	1.60821	1.41132
	Cumulative variance%	37.9	58.0	75.6

FLT: Flag leaf thickness, SLW: Specific leaf weight, SPT: Spike thickness, SPL: Spike length, q_N_: Non-phochemcial quenching, QY_max: Maximum quantum yield of PSII, Roundness: Grain roundness, SSPY: Single spike yield, DON: Deoxynivalenol.

## Discussion

Fusarium head blight, being an ancient and re-emerging disease, still poses major challenges to food crops, of which wheat is one of the important cereal crops affected by FHB and causing major crop losses. Various chemical control strategies are being employed in the cessation of FHB incidence, which are potential threats to the environment and climate change. The current investigation was aimed at controlling FHB infection by employing BCM, as an alternative strategy to conventional chemical control for sustainable agriculture. The discovery of new biocontrol agents was presented as an eco-friendly approach for integrated Fusarium management towards yield preservation and mycotoxin reduction in cereal crops. Previous reports of bacterial biocontrol agents against Fusarium disease were reported in wheat viz*., Clonostachys rosea*, *Sarocladium*, *Anthracocystis* and *Penicillium*^36,37^. In the present investigation, four strains of *Pseudomonas* and two strains of *Serratia* were used as consortia to control the FHB infection. The two Fusarium isolates used in the present study showed growth compatibility through plate assays through the formation of overlapping mycelial growth on the growth medium. In vitro plate assay for antagonistic activity of BCM consortia is evident from 35% (VOCs), 24% (NVOCs) and 81.6% (mycelial dry weight) reduction in mycelial growth, indicating a potential biocontrol effect of BCM consortia on Fusarium. The growth inhibitory effects of *Pseudomonas* on Fusarium are executed through bacterial production of phenazine-1-carboxamide, pyrrolnitrin, which acts against FHB infection^38,39^. *Serratia marcescens* is one of the chitinolytic bacteria comprising two chitinase genes viz*., chi*A and *chi*B, which act on the β-1,4 glycosidic linkages of cell wall chitin, resulting in the destruction of cell wall structural integrity and leading to a growth inhibitory effect on Fusarium mycelia^40^.

Wheat spikes inoculated with Fusarium spores (FHB treatment) started exhibiting symptoms from 2 dpi, whereas treating the plants with BCM (FHB + BCM treatment) resulted in delayed onset of symptoms, indicating the reduced severity of the FHB pathogen. Among all the inoculated spikes in FHB + BCM treatment, only 33% spikes showed the development of initial symptoms within the spike, which corroborates the likelihood of the development of resistance to initial FHB infection. Point inoculation of individual spikelets resulted in the rapid spread of the Fusarium in FHB treatment, as evidenced by higher AUDPC (332.33), whereas the BCM consortia treated plants (FHB + BCM treatment) effectively curtailed the proliferation and spread of FHB and maintained lower AUDPC values (11.89), inferring BCM induced evoking of type-II resistance for FHB in wheat. Biocontrol triggers alterations in the composition of cell wall constituents at the infection site, including lignin, pectin, and hemicellulose, thereby preventing cell plasmolysis and collapse, causing curtailed pathogen spread towards the development of type II resistance^41,42^. Besides type II resistance, the application of BCM also resulted in a significant reduction in grain DON accumulation (47%), eliciting type V resistance. SSPY had reduced under FHB treatment, however, application of BCM resulted in the sustenance of grain yield under FHB infection. Similar results were reported with *Bacillus* strains, evidencing the yield increment and reducing the severity of FHB disease^43^. Spike fluorescence characters such as F_m_, F_v_/F_m_, PSIIOPE, and QY_max were reduced under FHB infection (FHB treatment), which is mitigated with the application of BCM (FHB + BCM treatment). Application of BCM resulted in the sustenance of light-harvesting complex functioning for harvesting more light energy (Fm), leading to higher QY_max, F_v_/F_m_ and lesser photochemical quenching. Reduced Fm under FHB infection possibly resulted from inefficient reduction of quinone associated with reduced operational efficiency in light energy capturing^44^. Higher PSII operational efficiency (PSIIOPE) under FHB + BCM, accompanied by a greater number of open reaction centers (qL) further corroborates the BCM influence in maintaining the spike photosynthetic performance and mitigating FHB spread. The rate of steady-state fluorescence decay was higher under FHB infection (FHB treatment), and application of BCM (FHB + BCM treatment) reduced Rfd_Lss by 9%. Rfd_Lss symbolizes the rate of decline in steady-state fluorescence, a measure applicable for assessing the health of plants and indicating their photosynthetic efficiency^45^. The quenching parameters (q_P_ and q_N_) started to decline with the progression of FHB infection, which is probably due to the degree of biotic stress exceeding the limit of the thermal dissipation defense system in the spike resulting in failure of the photosynthetic process^46^. Grain morphology features and SPT significantly varied between treatments and exhibited significant reduction due to FHB infection (FHB treatment), but these changes were reversed when BCM were applied (FHB + BCM treatment), which could be attributed to the antagonistic action of BCM on FHB, resulting in efficient maintenance of grain length, width, mean width, volume, roundness and total area leading to better yield. Grain morphology features are crucial in determining grain weight and the phenotyping of wheat seeds with Cgrain is considered to be an efficient strategy in delineating FHB in wheat^47,48^. Application of BCM resulted in a significant increase in SLA and reduction in FLT, which might be associated with high tissue N concentrations with augmented rates of CO_2_ assimilation and N uptake per unit leaf and root mass resulting high photosynthetic N utilization^49^. Path analysis indicated that SSPY had large direct positive effects from SPT, QY_max and roundness. SSPY is positively correlated with grain roundness through grain width, volume and higher SSPY under FHB + BCM treatment is associated with maintenance of better roundness through higher grain width and volume (Fig. 5B). Similar results of significant positive association of grain width with thousand grain weight in wheat is reported earlier in wheat^50^. AUDPC and DON are negatively related with the spike fluorescence traits with direct negative effect from QY_max on the grain DON accumulation. Spike chlorophyll fluorescence evaluation is an precisely reliable indicator for spike health and any changes associated with it can be related to the Fusarium infection^51^. Higher QY_max and reduced accumulation of DON under FHB + BCM treatment further corroborate the healthier immune response of plants, and reduced pathogen invasibility with the application of BCM^35^. The increase in PSII efficiency with the application of BCM could be contributing to dispensing the metabolic demand of energy requirements to trigger a defense response against the FHB stress^52–54^.

PCA plot indicated that first PC correlated strongly with QY_max, roundness and SSPY, and the second PC is lined with SPL and QY_max, which infers that these traits vary together. The first PC is considered as a measure of spike fluorescence and grain morphology traits and the second PC can be inferred as a measure of spike morphology characters. Grain DON accumulation had the largest negative loadings from QY_max on PC1 and PC2, corroborating the importance of QY_max and its significant negative influence on FHB progression and DON accumulation.

## Conclusion

The current investigation of identifying potential bacterial consortium for improving wheat FHB resistance is established with newly identified BE strains of Pseudomonas and Serratia. Taken together, the data suggested that QY_max of spike is a reliable indicator for SSPY and BCM induced a significant reduction in AUDPC and DON. The newly identified BCM were proven to be eliciting type II and V resistance responses for Fusarium infection in spring wheat under controlled growth conditions, which needs to be further dissected at the molecular level to understand the genes and regulatory pathways involved in evoking disease resistance. QY_max identified in the present investigation with direct positive effects on SSPY and negative effects on grain DON content is inferring that sustenance of spike fluorescence could be the vital factor for both yield formation and disease resistance. The identified spike fluorescence traits might be translated into phenotyping tools for large scale screening of wheat germplasm towards enhanced breeding for FHB.

## Material and methods

### *Fusarium* inoculum preparation and infection

Two FHB isolates belonging to *F. graminearum* provided by the plant breeding company Lantmännen Lantbruk were used in the present study. The isolates were grown at 24 °C on Spezieller Nahrstoffarmer Agar media^55^, macroconidial formation was incited and inoculum of 5 × 10^5^ spores /ml was prepared with protocol published earlier^56^. A similar concentration (5 × 10^5^ spores /ml) of spore suspension was used for the plate assay and drop inoculation to spike. Drop inoculation was performed on four florets with 7 µl spore suspension droplet/floret, which is directly delivered to the center of florets with a fine syringe at anthesis stage.

### Fusarium compatibility and antagonistic activity assay

Compatibility between the two *F. graminearum* isolates was investigated according to the method described by^57,58^. The antagonistic assay was conducted through three tests viz*.,* (1) antibacterial activity of volatile compounds (VOCs), (2) non-volatile compounds (NVOCs), 3) PDA liquid medium culture. The antifungal activity of VOCs from the community of isolates was investigated as described by^59^. To investigate the biocontrol effects of NVOCs, each bacterial strain was cultured separately in a liquid LB medium and kept in the incubator for 48 h at 28 °C. After the growth period, 1 ml of 0.2 OD of each strain was transferred and mixed in a clean falcon tube. The bacterial suspension was then centrifuged at 4200 rpm for 15 min. Two mL of the supernatant was filtered through a 0.22-micron MilliPore (MP) filter, (Sarstedt, Nümbrecht, Germany) supplemented with 18 mL of a PDA medium in a 9:1 ratio, and placed in a Petri dish. After drying, 20 µl of spore solution of *F. graminearum* strains 1 and 3 were placed in the center of the PDA medium. The resulting petri dishes were maintained at a temperature of 28 °C. The radial growth of the fungal colony was measured, and its inhibition percentage was calculated using the previously reported Eq^60,61^.

### Ethical approval

We hereby declare that the current research work does not involve any species at risk of extinction or endangered species.

### Greenhouse evaluation

#### Plant material and study system

One spring wheat genotype SW151002 (*Triticum aestivum* L.) was grown in a randomized complete block design with three replications, constituting 18 plants per each replication in biotron (a controlled climate chamber facility with adjustable climate conditions at the Swedish University of Agricultural Sciences, Alnarp, Sweden) by employing standard irrigation and fertigation practices. To ensure consistency in the experimental process, only plants that exhibited uniform spike emergence were selected for these experiments. The spikes of these selected plants were then tagged to facilitate precise inoculation and subsequent evaluation. Growing conditions are maintained as 16/8 h (day/night time) at 24 °C with a relative humidity of 60%. Seeds were germinated in petri dishes and three seedlings were transplanted per pot filled with peat soil from Emmalikunga Torvmull AB, Sweden. Four treatments were maintained to evaluate the effect of bacterial consortium on curtailing Fusarium infection viz*.,* 1) control (drop inoculation of sterile water), 2) Bacterial consortium—BCM (sterile water droplet inoculation + plants treated with bacterial consortia), 3) Fusarium inoculation—FHB (drop inoculation of Fusarium spores), 4) Fusarium inoculation with bacterial consortium treatment—FHB + BCM (plants treated with bacterial consortia + drop inoculation of Fusarium spores).

#### Bacterial treatment and FHB conditions

A group of six bacterial isolates viz*., Pseudomonas tolaasii-1523c, Serratia marcescens-2′39f., P. tolaasii-15.1e, P. tolaasii-15.1c, P. tolaasii-15.7 and S. marcescens-1523a* (GenBank ID of the bacterial isolates were provided under Supplementary Table S1), isolated from wild finger millet were evaluated against *F. graminearum* isolates. Bacterial consortia, at a concentration of 0.2 Optical Density (OD) for each strain, were applied to the plants during three key growth stages leading up to flowering. Specifically, the application was as follows: at 15 days after sowing (DAS), 25 ml per pot were applied through soil flooding; at 28 DAS, 30 ml per pot were introduced via both soil flooding and foliar spraying; and finally, at 45 DAS, 100 ml per pot were distributed through both soil flooding and foliar spraying. This application schedule was designed to ensure optimal uptake and efficacy of the bacterial consortia in enhancing plant resistance and health at crucial developmental phases. Plants bearing spikes on their main tillers, with similar anthesis stages, were tagged with markers before the day of inoculation procedures. Fusarium suspension at the rate of 5 × 10^5^ spores /ml was drop inoculated to four spikelets per spike (7 µl /spikelet) of the main tiller with a fine needle for FHB and FHB + BCM treatments, whereas the control and BCM treatments were inoculated with sterile water droplets during anthesis stage of the plants. After the drop inoculation, the plants were maintained at 90% RH while keeping other parameters unchanged for 48 h to facilitate the growth of Fusarium. The visual scoring of FHB development on the spikes was carried out on 2, 3, 4, 5, 7, 9, 11, 13 days post inoculation (dpi) by observing for the development of bleached, yellowish, discolored, shriveled spikelets, indicating the presence or absence of disease on the spikes and severity of the FHB was assessed by employing 0 to 100% scale^62,63^.

#### Phenotypic measurements

Spike chlorophyll fluorescence measurements are executed with FluorCAM 700MF, PSI, Brno, Czech Republic, with measuring sequences of fluorescence images with a user-defined timing of set points, measurement intervals, and irradiance. Basic fluorescence F0 was induced by two panels of super-bright orange light emitting diodes (λmax = 620 nm, 345 LED per panel; approx. 3 μmol m^−2^ s^−1^). Maximum fluorescence (F_m_) was triggered by short-term (1 s) saturation light pulses (max. 2,500 μmol photons m^−2^ s^−1^) generated by an electronic shutter-equipped halogen lamp (250 W). Plants were dark adapted for 15 min and each measurement lasted 90 secs and is performed on the same side of intact spikes by bending the pot to 90°. Spike fluorescence generated fluoresence maximum (F_m_), minimum (F_o_), variable (F_v_), quantum yield of PSII (F_v_/F_m_), fraction of open reaction centers (qL), quenching parameters (non-photochemical quenching—q_N_, photochemical quenching—q_P_), maximum PSII quantum yield (QY_max), fluorescence decline ratio (Rfd) and in parallel the operating efficiency of PSII (PSIIOPE) was calculated as F′_q_/F′_m_, which infers the efficiency of PSII antennae towards utilizing the absorbed light in photochemistry^64^.

Leaf area at the anthesis stage was measured using LI-3000C portable leaf area meter (LI-COR Environmental, USA). Leaf thickness (LT) during the anthesis stage was measured with digital calipers and expressed in mm (Article number: 77001, Goobay®, Wentronic GmbH, Germany). Specific leaf area (SLA) and specific leaf weight (SLW) were calculated from the leaf area and leaf dry weight.

During the physiological maturity stage, the inoculated spikes were harvested and spike dimensions such as spike length (SPL) in centimeters and spike thickness (SPT) in millimeters were measured with digital calipers. Grains were threshed from the spike and the grain morphology traits were measured for the grains with a commercially available imaging instrument called Cgrain Value™^65^, which gives out 26 parameters, of which eleven attributes are relevant to the present investigation viz*.,* area, length, width, thickness, mean width, volume, weight, light, hue, saturation and roundness of the grains. During physiological maturity, the tagged spike is harvested, threshed and total weight of grains per spike is reported as single spike yield (SSPY) in grams.

#### DON assay

DON accumulation in the grain was estimated with commercially available enzyme-linked immunosorbent assay (ELISA, RIDASCREEN FAST DON, R-Biopharm AG). One gram of finely ground grain samples was used in 20 ml of sterile distilled water for extracting DON, following standard guidelines provided by the manufacturer. A microplate spectrophotometer (Thermo Scientific™ Multiskan™ FC Microplate Photometer) was used to record the absorbance at 450 nm and the results were analyzed with RIDA SOFT Win.net provided by the manufacturer. DON content is expressed in ppm.

Statistical analysis: Statistical analyses were conducted using R^66^ and Statistical Tool for Agricultural Research (STAR) Version: 2.0.1, International Rice Research Institute (IRRI), Philippines. Area under disease progress curve values (AUDPC) of Fusarium disease progression and area under curve (AUC) values of spike fluorescence traits were calculated with Agricolae in R^67^. AUDPC was calculated by evaluating the disease progression with appearance of symptoms on inoculated spike of each plant from 2 to 8dpi (2, 3, 4, 5, 7, 9, 11, 13dpi), and the calculation was done based on following equation with audpc function in agricolae package of R^68^.

$${\text{AUDPC}} = \mathop \sum \limits_{i}^{n - 1} \left( {\frac{{y_{i} + y_{i + 1} }}{2}} \right)\left( {t_{{{\text{i}} + 1}} - t_{i} } \right)$$

where y_i_ is disease score in the ith date, t_i_ is the ith day, n is the number of dates on which disease was recorded.

Path coefficient analysis was performed by employing correlation analysis according to Dewey and Lu^69^ and direct and indirect effects were calculated as described by Akintunde^70^. To account for multicollinearity, the phenotypic traits with variance inflation factor (VIF) values less than two were selected for path coefficient and PCA analysis. Based on VIF values, seven traits viz*.,* FLT, SLW, SPT, SPL, q_N_, QY_max, roundness were selected as depicted in Supplementary Table S2. The analysis for path coefficeint and PCA were executed separately for uninfected (combined control and BCM treatments) and infected conditions (combined FHB and FHB + BCM treatments) to understand the physiological effects on the SSPY and FHB associated DON accumulation.

### Supplementary Information

Supplementary Table S1.

Supplementary Table S2.

## Data Availability

The Sanger sequencing data of bacterial isolates has been deposited at NCBI GenBank and the accession numbers and sequence details are provided under Supplementary Table S1. The datasets generated and/or analyzed during this study are included in the article and supplementary material.
