# Supplementary material for: A new bacterial consortia for management of Fusarium head blight in wheat
Source: Sci Rep. 2024 May 2;14:10131. doi: 10.1038/s41598-024-60356-4 (PMC11066059; doi:10.1038/s41598-024-60356-4)
Supplement: Supplementary file 1 — Supplementary Table S1. [file 41598_2024_60356_MOESM1_ESM.docx]

Supplementary Table S1: GenBank ID for bacterial consortia employed in the current investigation

| **SNO** | **Organism** | **GenBank ID** |
| --- | --- | --- |
| 1 | *Pseudomonas tolaasii*-1523c | PP386803 |
| 2 | *Serratia marcescens*-2'39f | PP386806 |
| 3 | *Pseudomonas tolaasii*-15.1e | PP386807 |
| 4 | *Pseudomonas tolaasii*-15.1c | PP386808 |
| 5 | *Pseudomonas tolaasii*-15.7 | PP386809 |
| 6 | *Serratia surfactantfaciens*-1523a | PP440168 |
